# The frequency of regulatory T-cells in Hashimoto’s thyroiditis and Graves’ disease

**DOI:** 10.1007/s10238-025-01964-w

**Published:** 2025-11-27

**Authors:** Reham Mohamed El Shabrawy, Zeinab Ahmed Ashour, Fawzia Abo Ali, Salwa Seddik Hosny, Nermin Melek, Ashraf Okba

**Affiliations:** 1 Microbiology and Immunology Department, Faculty of Medicine, Zagazig University, Zagazig, Egypt; 2 Internal medicine, Allergy and Clinical Immunology department, Faculty of Medicine - Ain Shams University, Cairo, Egypt; 3 Internal Medicine and Endocrinology, Faculty of Medicine, Ain Shams University, Cairo, Egypt

**Keywords:** T-regulatory cells, FOXP3, Autoimmune thyroiditis, Hashimoto thyroiditis, Graves’ disease

## Abstract

Till now, the management of autoimmune thyroid diseases (AITD) depends on symptomatic treatment and replacement or anti-thyroid therapy. Uncovering the pathophysiologic mechanisms of autoimmunity provides hope for new insights into management. These new treatments aim to modulate the immune reaction and stop the autoimmune process. T regulatory cells (Tregs) are central in antagonising autoimmunity. This study aimed to compare the number of CD4/CD25/FOXP3 T regulatory cells in the different forms of autoimmune thyroid diseases and in the normal population, and to compare the number of CD4 + CD25 + FOXP3 + T regulatory cells between the different forms of AITD, HT, and GD. Also, to investigate the difference in the number of CD4/CD25/FOXP3 T regulatory cells in AITD associated with allergic disorders on one hand and autoimmune thyroid diseases not associated with allergic disorders on the other hand. This study included 18 patients suffering from Hashimoto’s thyroiditis (HT), 15 patients suffering from Graves’ disease (GD); and, for comparison, the Tregs level was measured in 15 healthy control patients. A statistically significant decrease was found regarding CD4/CD25, CD25/FOXP3 percentages and CD4/CD25/FOXP3 absolute number between patients of AITD and the normal population. The absolute number of CD4/CD25/FOXP3 was lower in the GD group than in HT group. Allergic comorbidities do not influence Tregs percentage or their CD4/CD25/FOXP3 absolute number in any of the AITD forms. Tregs may be a potential therapeutic target for AITD.

## Introduction

Hashimoto’s thyroiditis (HT) and Graves’ disease (GD) represent diverse forms of autoimmune thyroid diseases; HT is a chronic thyroid inflammation that affects women predominantly. It is regarded as the most common organ-specific autoimmune disease. It is characterized by follicular lymphocytic infiltration in the thyroid gland with the development of anti-thyroid peroxidase enzyme (Anti-TPO) and/or anti-thyroglobulin (Anti-TG) [1].

Graves’ disease is an autoimmune disorder in which the body produces antibodies specific to the receptor for thyroid-stimulating hormone. These antibodies cause hyperthyroidism because they bind to the TSH receptor and chronically stimulate it. This, in turn, causes clinical symptoms of hyperthyroidism, and the enlargement of the thyroid gland is visible as a goiter [2].

Regulatory T cells (Tregs) are a subset of CD4 + T cells that comprise 5%– 10% of CD4 + T cells. These cells function through several mechanisms, including cell-to-cell contact and the production of immunosuppressive cytokines, such as transforming growth factor (TGF)-β and interleukin (IL)-10, which inhibit antigen-specific T-cell responses [3]. They are considered an important component in the understanding of the immune response to pathogens and the mechanisms of peripheral tolerance that control the development of allergies and autoimmune diseases. In mice and humans, Treg cells are characterized by high expression of CD4, CD25, and FOXP3. Studying the role of Tregs in autoimmune thyroid diseases may provide insight into the underlying mechanisms responsible for the development of autoimmune diseases, aiming to find a new therapeutic strategy that improves disease outcome [4]. While there is a growing number of studies investigating the role of Treg cells in autoimmune thyroid diseases in mice, studies on autoimmune thyroiditis in humans are still limited [5].

This study aimed to compare the number of CD4/CD25/FOXP3 T regulatory cells between the different forms of autoimmune thyroid diseases from one hand, and to compare the number of CD4 + CD25 + FOXP3 + T regulatory cells between the different forms of AITD and a control group on the other hand. Also to investigate if there is a difference in the number of CD4/CD25/FOXP3 T regulatory cells in AITD who suffer from allergic disorders and those who are not allergic.

## Subjects and methods

### Subjects’ characteristics

This study included 18 patients suffering from Hashimoto’s thyroiditis, 15 patients suffering from Graves’ disease; and, for comparison, the Tregs level was measured in 15 healthy control patients.

The inclusion criteria were as follows: informed signed consent of adult patient ( 18 years and older); patients diagnosed with AITD (HT or GD) based on symptoms, signs, clinical examination, and laboratory examination. To assess thyroid function, the serum concentration of TSH, FT3, and free FT4 were measured. For HT, the key diagnostic criterion was an elevated level of Anti-TPO and/or Anti-TG in a patient presenting with a goitre, reduced thyroid gland size (atrophic thyroid), or hypothyroidism. The diagnosis of GD was confirmed in patients with Hyperthyroidism and elevated thyroid receptor antibody levels. Patients were additionally subclassified into allergic and non-allergic groups. Clinically diagnosed allergic disorders include (e.g., asthma, allergic rhinitis, atopic dermatitis, or confirmed food allergy).

Exclusion criteria were: Patients with severe renal or liver impairment, patients who received immunosuppressive ( e.g. disease-modifying antirheumatic drugs) or corticosteroid medications during the last three months before the study, patients who received allergen immunotherapy or biological therapy, or patients who received radioactive iodine, patients with thyroidectomy, pregnant or breastfeeding female patients, and patients suffering from concomitant autoimmune disease. Also, patients who suffer from HIV or any active acute infection at the time of blood sampling, or those with a history of malignancy or chemotherapy, were excluded from the study.

### Case definition

All healthy individuals were subjected to full clinical examination, measurement of Thyroid function test (TSH, free T3, free T4) and detection of the frequency of CD4/CD25/FOXP3 Tregs using flow cytometry.

Diagnosis of autoimmune thyroiditis was based on symptoms, signs, and clinical examination. Thyroid function test (TSH, free T3, free T4), detection of autoantibodies (anti-TPO, anti-TG, anti-TSHR) and measuring CD4/ CD25/FOXP3 Tregs using flow cytometry.

The diagnosis of HT was confirmed in patients with elevated levels of TPO and anti-TG antibodies in a patient who presents with a goiter, or reduced size of the thyroid gland (atrophic thyroid), or hypothyroidism. The diagnosis of GD is confirmed in patients with Hyperthyroidism and elevated anti-TSHR, or isolated thyroid-associated orbitopathy with elevated thyroid receptor antibody levels [6].

### Laboratory investigations

Thyroid function tests, the serum levels of free T3 (FT3), free T4 (FT4), and thyroid-stimulating hormone (TSH), were measured by electrochemiluminescence on Cobas 6000 analyzer (Roche Diagnostic, USA).

To measure anti-TPO, anti-TG and anti-TSHR, we used three different kits of a double-antibody sandwich enzyme-linked immunosorbent assay (ELISA) specified to assay human serum level of anti-TPO, anti-TG and anti-TSHR (My biosource-kit, USA) according to the manufacturer’s instructions.

Detection of CD4/CD25/FOXP3 Tregs by flow cytometry (FACSCaliber 4 colour, Becton Dickinson, San Joe, USA) was done using a mixture of monoclonal antibodies added directly to peripheral blood samples according to the manufacturer’s instructions. The monoclonal antibodies used included CD4 FITC, CD25 4E3 and FOXP3 FITC (supplied by Invitrogene, Thermofisher, USA). CD4 and CD25 were detected using surface staining technique, while FOXP3 was detected by intracellular staining.

### Statistical analysis

We used the Statistical Package for the Social Sciences for Windows (version 21.0; SPSS Inc., Chicago, IL, USA).

## Results

### Patients’ characteristics

Subjects included in the study showed no statistically significant difference regarding sex and age. The mean age of patients included in this study was 47.9 ± 13.5 for the control group, 45 ± 13.3 for the GD and 45.2 ± 17.4 for HT patients’ group.

### HT patients’ group

Regarding the HT group, the most common presentation was hair loss, followed by fatigue, the mean ± SD TSH serum concentration among them was 27.6 ± 22.5 uIU/L (normal: 0.5–4.3 uIU/ml), Free T3 was 2.8 ± 0.9 (normal: 0.9 to 2.8 pg/mL), while freeT4 was 3.5 ± 6.9 (normal: 0.73–1.43 ng/dL), the mean ± SD of anti TPO among patients with Hashimoto’s thyroiditis was 292.6 ± 296.2 (normal: <34 IU/mL), and the mean of anti-TG was 201.7 ± 76.9 (normal: <155 IU/mL). The mean percentage of CD4/CD25% and CD25/FOXP3% were 0.47 ± 0.1, and 19.1 ± 15.2 respectively. The mean ± SD of the absolute count of CD4/CD25/FOXP3 was 44.3 ± 10.8 cell/ul.

We found a highly significant positive correlation between CD4/CD25% and FT3 (*r* = 0.791, *p* = 0.001). CD25/FOXP3% shows a significant positive correlation with both FT3 (*r* = 0.756, *p* = 0.002) and FT4 (*r* = 0.585, *p* = 0.017). No significant correlation was found between CD25/FOXP3% and TSH. Also, a significant negative correlation was found between CD4/CD25 and anti-TG (-*r* = 0.969, *p* = 0.007).

When comparing with the control group, we found a statistically significant difference between both groups regarding the percentage of CD4/CD25 cells and a highly significant differences were found when the percentage of CD25/FOXP3 and the absolute count of CD4/CD25/FOXP3 cells. (Table 1).

**Table 1:** Comparison of Treg between the control group and HT patients group

	Studied groups		t- test	*p*- value
	Control (*n* = 15)	Hashimoto’s (*n* = 18)		
	Mean ± SD	Mean ± SD		
CD4%	42.4 ± 7.5	44 ± 14.2	− 0.3564	0.72 NS
CD4/CD25%	0.67 ± 0.2	0.47 ± 0.1	− 2.5964	0.02 S
CD25/FOXP3%	44.3 ± 15.2	19.1 ± 15.2	− 5.2402	< 0.001HS
CD4/CD25/FOXP3 (no/ul)	65.6 ± 6.7	44.3 ± 10.8	− 7.8887	< 0.001HS

*p* > 0.5 NS = non-significant; *p* < 0.5 significant; *p* < 0.001 HS = highly significant

### GD patients’ group

Concerning patients with GD, the most common presentation was weight loss (73%), followed by palpitation and tremors (66.6%) each. The mean TSH serum concentration was 0.076 ± 0.14 mIU/L (normal: 0.5–4.3 uIU/ml), Free T3 was 40.8 ± 114.4 (normal: 0.9 to 2.8 pg/mL), while freeT4 was 59.9 ± 159.14 (normal: 0.73–1.43 ng/dL). The mean ± SD anti-TSHR was 13.5 ± 14.7 (normal: <1.22 Iu/L), and the mean ± SD percentage of CD4/CD25%, and CD25/FOXP3% were 0.62 ± 0.7 and 34.9 ± 10.2 respectively. The mean ± SD of the absolute count of CD4/CD25/FOXP3 was 44.3 ± 10.8 cell/ul.

There was a significant negative correlation between CD4/CD25 and both T3(*r*=-0.67, *p* = 0.023) and T4 (*r*=-0.681, *p* = 0.01). Also, there was no significant correlation between Tregs and Anti-TSHR.

A statistically significant difference was found between the percentage of CD4/CD25 cells and the percentage of CD25/FOXP3, when we compared the GD group and the control group. Meanwhile, a highly significant differences were found with the absolute count of CD4/CD25/FOXP3 cells. (Table 2).

**Table 2:** Comparison of Treg between the healthy control and GD patient group

	Control (*n* = 15)	Graves’ (*n* = 15)		
	Mean ± SD	Mean ± SD		
CD4%	42.4 ± 7.5	37.7 ± 10.5	0.960	0.175 NS
CD4/CD25%	0.67 ± 0.2	0.42 ± 0.9	− 3.06	0.003 S
CD25/FOXP3%	44.3 ± 15.2	20.7 ± 8.3	− 3.9883	0.002 S
CD4/CD25/FOXP3	65.6 ± 6.7	34.9 ± 10.2	− 8.8517	< 0.001HS

*p* > 0.5 NS = non-significant; *p* < 0.5 significant; *p* < 0.001 HS = highly significant

### Comparison of Tregs between groups included in the study

**Fig. 1:**
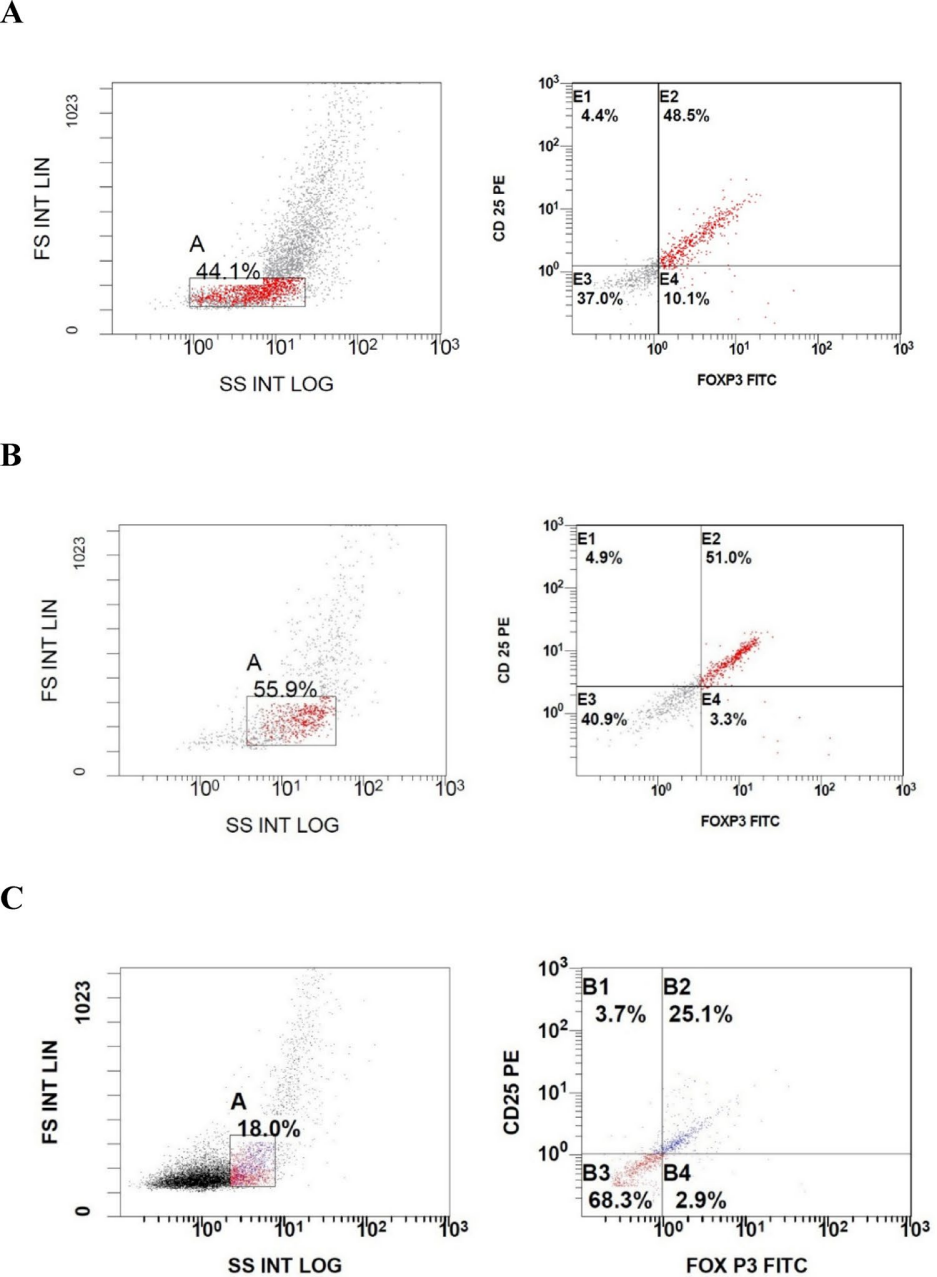
Difference in the percentage of CD25/FOXP3 between the three groups (**A**) A healthy control subject, (**B**) a case of Hashimoto’s thyroiditis, (**C**) a case of Graves’ disease

A highly statistically significant difference was found between the level of CD4+/CD25+%, CD4/CD25**/**FOXP3 control group, GD patients, and HT patients. Table 3, Figs. 1.

**Table 3:** Regulatory T cells comparison between the three studied groups

Variables	Studied groups			Test of sig.	*P*
	Control (*n* = 15)	Graves’ (*n* = 15)	Hashimoto’s (*n* = 18)		
	Mean ± SD Median	Mean ± SD Median	Mean ± SD Median		
CD4%	42.4 ± 7.5	37.7 ± 10.5	44 ± 14.2	F = 1.385	0.261 NS
CD4/CD25%	0.67 ± 0.2	0.42 ± 0.9	0.47 ± 0.1	KW = 10.713	0.005 S
CD25/FOXP3%	44.3 ± 15.2	20.7 ± 8.3	19.1 ± 15.2	KW = 21.88	< 0.001 HS
CD4/CD25/FOXP3	65.6 ± 6.7	34.9 ± 10.2	44.3 ± 10.8	F = 40.823	< 0.001 HS

KW = Kruskal-Wllis test; F = value of ANOVA test ; *p* > 0.5 NS = non-significant; *p* < 0.5 S = significant; *p* < 0.001 HS = highly significant

### Comparison of Tregs between HT and GD groups

When comparing Tregs between GD than in HT, there were significantly lower absolute count of CD4/CD25/FOXP3 in GD than in HT. However, no significant difference was found in the percentage of Tregs. (Table 4).

**Table 4:** Comparison between hashimoto’s thyroiditis and graves’ disease regarding regulatory T-cell

	Hashimoto’s thyroiditis Mean ± SD	Graves’ disease Mean ± SD	t-independent test	*p*- value
CD4%	44.04 ± 14.2	37.7 ± 10.5	1.353	0.093 NS
CD4/CD25%	0.47 ± 0.1	0.42 ± 0.9	1.091	0.142 NS
CD25/FOXP3%	19.1 ± 15.2	20.7 ± 8.3	0.363	0.359 NS
CD4/CD25/FOXP3	44.3 ± 10.8	34.9 ± 10.2	2.417	0.011 S

*p* > 0.5 NS = non-significant; *p* < 0.5 S = significant

### Tregs in allergic patients with AITD

Although the number of allergic patients in HT (8 out of 18) is higher than that of GD (2 out of 15), the difference was statistically insignificant. Additionally, no significant differences were found between allergic and non-allergic patients regarding thyroid functions, antibodies, and regulatory T-cells, among either the HT or the GD group. (Tables 5 and 6)

**Table 5:** Association between thyroid functions, antibodies, and regulatory T-cells and allergic status among the hashimoto’s disease group

Variables	Studied groups		Test of sig.	*P* value
	Not allergic (*n* = 10 )	Allergic (*n* = 8)		
	Mean ± SD	Mean ± SD		
T3	2.9 ± 0.9	3.1 ± 0.9	*t* = 0.297	0.77 NS
T4	3.3 ± 6.9	1.9 ± 1.6	MW = 35.0	1.0 NS
TSH	10.8 ± 16.6	22.6 ± 26.1	MW = 25.0	0.49 NS
Anti TPO	308.7 ± 342	260.5 ± 202	MW = 24.0	0.90 NS
Anti TG	155.3 ± 35.0	248 ± 84.4	*t* = 1.756	0.18 NS
CD4%	44.2 ± 12.9	43.9 ± 17.6	*t = 0.037*	0.97 NS
CD4/CD25%	0.49 ± 0.1	0.45 ± 0.1	*t* = 0.444	0.66 NS
CD25/FOXP3%	19.5 ± 8.3	22.2 ± 8.5	*t* = 0.635	0.535NS
CD4/CD25/FOXP3	39.9 ± 10.4	49.9 ± 9.1	*t* = 2.0	0.06 NS

MW = Mann-Whitney; T = Independent t test; *p* > 0.5 NS = non-significant

**Table 6:** Association between thyroid functions, antibodies, regulatory T-cells, and allergic status among GD group

Variables	Studied groups		Test of sig.	*P* value
	Not allergic (*n* = 13 )	Allergic (*n* = 2)		
	Mean ± SD	Mean ± SD		
T3	48.3 ± 126.5	7.3 ± 6.9	MW = 9.0	1.00 NS
T4	10.9 ± 25.5	290.9 ± 410	MW = 11.0	0.85 NS
TSH	7.1 ± 15.1	0.02 ± 0.01	MW = 5.0	0.232 NS
Anti TSH	12.5 ± 14.8	26.6 ± 0.7	MW = 4.0	0.142 NS
CD4%	39.1 ± 10.7	28.9 ± 2.9	T = 1.311	0.214 NS
CD4/CD25%	0.65 ± 0.7	0.44 ± 0.2	MW = 12.0	1.00 NS
CD25/FOXP3%	19.6 ± 16.3	15.7 ± 7.8	MW = 9.0	0.583 NS
CD4/CD25/FOXP3	34.9 ± 11.02	34.7 ± 2 0.7	T = 0.034	0.97 NS

MW = Mann-Whitney; T = Independent t test; *p* > 0.5 NS = non-significant

## Discussion

Till now, the management of AITD depends on symptomatic treatment and replacement or anti-thyroid therapy. Uncovering the pathophysiologic mechanisms of autoimmunity proves hope for new insights for management. These new treatments aim to modulate the immune reaction and stop the autoimmune process. Tregs are central in antagonizing autoimmunity [7].

The most common manifestations for HT patients included in this study were hair loss (83.3%), fatigue (77.8%), followed by cold intolerance (61.1%), muscle cramps, and weight gain. These findings are common for hypothyroidism [8]; however, different studies may show differences in the frequency of each symptom according to the studied population, the sample size, and the stage at which patients were diagnosed, whether subclinical or overt hypothyroidism [9]. However, fatigue, weight gain, trouble tolerating cold, joint and muscle pain were the first listed symptoms according to other authors [10].

The mean TSH serum concentration among patients of symptomatic HT was normal, while the detected levels of T3 and T4 were within normal. TSH is a sensitive test of thyroid function that is also elevated in subclinical hypothyroidism and is usually the initial laboratory abnormality detected as the pituitary gland attempts to increase thyroid hormone production from the failing thyroid gland. The total T4 or free T4 usually remains within reference ranges in subclinical hypothyroidism [11].

No association was found between any of the clinical presentations on one hand and any of the subsets of cells. Regarding thyroid function tests, we found that a decrease in the frequency of T regulatory cells was associated with a decrease in the level of FT3. In the work of Hu et al., Tregs showed an inverse correlation to thyroid function states in Hashimoto thyroiditis [12]. These findings illustrate that the decrease in the regulatory T cell frequency enhances the immune destruction state of thyroiditis and thus deteriorates the thyroid profile.

In this study, the percentage of CD4/CD25 is negatively correlated with the level of anti-thyroid antibodies (Anti-TG), while no correlation was found with the anti-TPO. To the best of our knowledge, the relation between anti-TG and Tregs has not been investigated in any other study yet, however, other authors found a negative correlation between the level of Treg cells CD4/CD25 and anti-TPO and concluded that this finding explains how the decrease in the Tregs contributes to thyroid immune damage in HT [13]. Treg cells play a crucial role in preventing the onset and progression by suppressing the production of harmful autoantibodies targeting TG and TPO by autoreactive B cells, thus preventing the destruction caused by these autoreactive B cells. Understandably, a low level of Tregs is associated with the development of Hashimoto’s thyroiditis [14, 15].

Comparing Tregs between the control group and Hashimoto’s disease, A statistically significant difference in the percentage of CD4/CD25 cells between the two groups was found. Meanwhile, highly significant differences were found when the percentage of CD25/FOXP3 and the absolute count of CD4/CD25/FOXP3 cells.

Our results were supported by Mazzieri et al., who stated that compared to healthy controls, HT patients showed a significant decrease in peripheral circulating FOXP3 Tregs [15]. Other researchers reported a similar result, finding that the number of CD4/CD25/FOXP3 Tregs was significantly lower in HT patients compared to the normal population [16–18].

On the other hand, Şıklar et al. found no difference in the frequency of Tregs in pediatric patients suffering from Hashimoto thyroiditis [19]. Interestingly, Guo et al. found that the proportions of peripheral Treg cells in patients with HT were significantly higher than in control subjects. They explain this finding by a compensatory attempt to overcome or reduce the autoimmunity by accelerating Treg cell activity [20].

In the population studied, the most common presentation among patients with Graves’ disease was weight loss (73%), followed by palpitation and tremors (66.6%) each. In another study, the most common symptoms of GD were palpitation (57%, followed by heat intolerance 53%, weight loss 53% and palpitation 49% [21, 22]. Different studies may show differences in the frequency of each symptom according to the studied population and the sample size.

Our result regarding serum levels of TSH, T3 and T4 represents the classical thyroid function profile in Graves’ disease as in overt hyperthyroidism, both serum free T4 and T3 concentrations are elevated, and serum TSH is suppressed [23].

A statistically significant negative correlation was found between the level of CD4/CD25 and both T3 and T4, indicating the relation between the level of Treg cells and the disturbance that occurs in the thyroid function abnormalities in cases of thyrotoxicosis [24]. Similarly, Zhong et al. found an inverse relationship between Tregs and T3 in patients with GD [25]. Although a negative correlation was found between the CD4/CD25 and anti-TSHR antibodies, the correlation was not statistically significant. This can be explained by the small sample size. However, Bossowski et al. found a statistically significant correlation between TSHR-Ab levels and the percentage of CD4 + IL-17+/CD4/CD25/CD127/ FOXP3 T cells [26]. Similarly, Teniente-Serraet al. reported a lower proportion of Tregs in the peripheral blood of GD patients and a negative correlation between anti-TSHR levels and the frequency of Tregs in GD patients [27]. Tregs’ depletion may be associated with the production/persistence of anti-TSHR [24].

When comparing Tregs in GD with the control group, we found a statistically significantly lower percentage of CD4/CD25 cells and CD25/FOXP3 in the GD GROUP than in the control group. Meanwhile, highly significant differences were found with the absolute count of CD4/CD25/FOXP3 cells.

The research work of Bossowski et al., found considerably lower rates of CD4 + CD25 + FoxP3 + in GD patients compared to healthy controls. However, they found no differences in the frequency of CD4 + CD25 T cells [26]. Decreased frequency of circulating CD4 + CD25 + FoxP3 + Tregs was also the conclusion of other researchers [18, 28, 29]. Decreased frequency of both circulating and intrathyroidal CD4 + CD25+ (both CD69 + and FoxP3+) cells was observed in other studies [29]. Deeper understanding came from a meta-analysis of six studies revealing a reduced frequency of circulating Tregs (identified as CD4 + CD45RA+, CD4 + CD25+/high, CD4 + CD25 + FOXP3+/high, CD4 + CD25 + CD69−) in untreated GD patients (sample size 258 patients) compared to matched healthy control [30].

On the other hand, Glick et al. found no significant differences in the frequency of CD4 + CD25high T cells as a percentage of CD4 + T cells between AITD patients (6.1% ± 0.4%) and healthy controls (6.5% ± 0.4%; *p* = 0.18) [31]. The same result was obtained by Cagiltay et al., who found no difference in the proportion of CD4 + CD25 + T cells [32].

A completely different finding was observed by Marazuela et al., who observed increased frequency of CD4 + lymphocytes expressing FoxP3, IL-10, and TGF-β in GD patients, which was associated with defective suppressive Tregs’ function [33].

As discussed before, studies that compared different forms of AITD with the healthy population yielded controversial results. In this study, when we compared the frequency of CD4/CD25/FOXP3 between the normal population and patients with AITD, we found that it was much lower in patients with AITD than in the normal population, highly statistically significant (*p* < 0.001, *p* < 0.005), respectively. Many factors can stand behind these variations of results. Firstly, the fact that circulating Tregs may not always correspond to intrathyroidal infiltrate, more research work is needed to correlate the frequency of circulating Tregs with the amount of intrathyroidal Tregs. Additionally, the individual functions of Tregs should be thoroughly investigated, as an increased number in the absence of adequate function may have the same effect as fewer of these cells. Also, the presence of certain Tregs inhibitors may affect the function in vivo, as indicated by Rodríguez-Muñoz and colleagues, who found that circulating Microvesicles from AITD patients have a functional role and can inhibit Treg differentiation [34]. The presence of distinctive Tregs phenotypes, as indicated with different markers e.g., (FOXP3, CD127, GITR) may also explain the result variations. Gho et al. followed two cohort studies: a European cohort study that identified five Treg cell phenotypes that causally protect against HT risk, and another Asian cohort study that identified four Treg cell phenotypes negatively correlated with the risk of HT [35].

According to our results, the frequency of CD4/CD25/FOXP3, but not CD4+/CD25 + in Graves’ disease was lower than Hashimoto’s thyroiditis. This result may indicate that certain Treg phenotypes may play different role in different autoimmune diseases. After reviewing the literature, to our knowledge, this is the first study comparing the frequency of Tregs in both diseases. Studying different aspects of the autoimmune process and different populations of Tregs in both diseases is needed to approve or disapprove this finding.

When we compared the difference between the Treg cell frequency in both the allergic and non-allergic groups, we found no statistically significant difference between them. Probably because the level of Tregs in those patients is already reduced, and that this reduction is sufficient for causing both conditions in susceptible patients.

## Conclusion

In summary, a statistically significant decrease was found regarding CD4/CD25, CD25/FOXP3 percentages and CD4/CD25/FOXP3 absolute number between patients of AITD and the normal population. The absolute number of CD4/CD25/FOXP3 was lower in the Graves’ disease group than in Hashimoto’s thyroiditis group. Allergic comorbidities do not influence Tregs percentage or their CD4/CD25/FOXP3 absolute number in any of the AITD forms. Given the limitations of currently available AITD treatments, identifying potential pathogenetic factors for pharmacological targeting is of paramount importance. The proven frequency reduction of Tregs in patients with AITD marks Tregs as an excellent candidate for a novel diagnostic tool as well as biological immunotherapy.

## Data Availability

The datasets used and/or analyzed during the current study are available from the corresponding author upon reasonable request.
